# Chemical named entity recognition in patents by domain knowledge and unsupervised feature learning

**DOI:** 10.1093/database/baw049

**Published:** 2016-04-16

**Authors:** Yaoyun Zhang, Jun Xu, Hui Chen, Jingqi Wang, Yonghui Wu, Manu Prakasam, Hua Xu

**Affiliations:** ^1^School of Biomedical Informatics, University of Texas Health Science Center at Houston, Houston, TX 77030, USA; ^2^School of Biomedical Engineering, Capital Medical University, Beijing 100069, China; ^3^Mira Loma High School, Sacramento, CA 95821, USA

## Abstract

Medicinal chemistry patents contain rich information about chemical compounds. Although much effort has been devoted to extracting chemical entities from scientific literature, limited numbers of patent mining systems are publically available, probably due to the lack of large manually annotated corpora. To accelerate the development of information extraction systems for medicinal chemistry patents, the 2015 BioCreative V challenge organized a track on Chemical and Drug Named Entity Recognition from patent text (CHEMDNER patents). This track included three individual subtasks: (i) Chemical Entity Mention Recognition in Patents (CEMP), (ii) Chemical Passage Detection (CPD) and (iii) Gene and Protein Related Object task (GPRO). We participated in the two subtasks of CEMP and CPD using machine learning-based systems. Our machine learning-based systems employed the algorithms of conditional random fields (CRF) and structured support vector machines (SSVMs), respectively. To improve the performance of the NER systems, two strategies were proposed for feature engineering: (i) domain knowledge features of dictionaries, chemical structural patterns and semantic type information present in the context of the candidate chemical and (ii) unsupervised feature learning algorithms to generate word representation features by Brown clustering and a novel binarized Word embedding to enhance the generalizability of the system. Further, the system output for the CPD task was yielded based on the patent titles and abstracts with chemicals recognized in the CEMP task.

The effects of the proposed feature strategies on both the machine learning-based systems were investigated. Our best system achieved the second best performance among 21 participating teams in CEMP with a precision of 87.18%, a recall of 90.78% and a *F*-measure of 88.94% and was the top performing system among nine participating teams in CPD with a sensitivity of 98.60%, a specificity of 87.21%, an accuracy of 94.75%, a Matthew’s correlation coefficient (MCC) of 88.24%, a precision at full recall (P_full_R) of 66.57% and an area under the precision-recall curve (AUC_PR) of 0.9347. The SSVM-based CEMP systems outperformed the CRF-based CEMP systems when using the same features. Features generated from both the domain knowledge and unsupervised learning algorithms significantly improved the chemical NER task on patents.

Database URL: http:// database. oxfordjournals. org/ content/ 2016/ baw049

## Introduction

Chemical compounds and drugs mentioned in scientific text are crucial for many computational applications in the biomedical domain, such as drug repurposing (1) and construction of gene–chemical interaction pathways (2). In addition to the development of chemical/drug databases such as PubChem (3), ChEBI (4), ChemSpider (5) and DrugBank (6), extensive efforts have been applied for extraction of chemical information from rich textual sources such as biomedical literature. For example, the BioCreative IV Chemical and Drug Named Entity Recognition (CHEMDNER) challenge promoted the development of chemical NER systems for scientific literature, by providing a large**-**scale standard corpus (7). Typical approaches applied to chemical NER in representative systems such as Whatizit (8), OSCAR3/4 (9, 10), ChemSpot (11) and tmChem (12) included dictionary lookup (8), machine learning-based models (9, 10, 12) and hybrid methods that combined a dictionary with a machine learning-based classifier (11). In addition to a set of common NER features used widely in different genres, features generated from domain knowledge (12, 13) and unsupervised learning methods (14, 15) showed promising results in chemical NER for biomedical literature in the past.

Medicinal chemistry patents are another important source for text mining approaches to assist in drug development (16), which has been attracting increasing attention from the Pharma and Biotech industries in recent years (17). Compared with other biomedical texts such as clinical notes and scientific literature, patents have their own document formats, linguistic structures and terminologies (18). There are some previous studies that focus on chemical NER from patents (19–27). The chemical datasets annotated for patents were usually small (28), of which the most commonly used corpus contained only 40 patents released by ChEBI (19–23). To enlarge the training dataset for patent chemical NER, several studies leveraged the corpora from other resources, such as the CHEMDNER corpus built from biomedical literature (22, 23), and the DDI corpus (20, 23) built from both DrugBank and biomedical literature. As seen in literature, the major approaches for building chemical NER systems for patent text were dictionary lookup (19, 21, 25, 27) and machine learning**-**based methods using conditional random fields (CRF) algorithm (19–24, 29). Efforts were also made to validate the recognized chemicals using the semantic similarities between chemical pairs (22, 23). However, no comparative evaluation of different chemical NER systems has been conducted on a large annotated corpus of patents. Moreover, it is also not clear how different types of features contribute to the performance of chemical NER systems for patent documents.

To promote the development of NER systems for medicinal chemistry patents, the Spanish National Cancer Research Center (CNIO), Universidad Politecnica de Madrid and University of Navarra co-organized a challenge on CHEMDNER for patents (CHEMDNER patents), as a part of BioCreative V challenge (Track 2) (30). This challenge included three individual subtasks: (i) Chemical Entity Mention Recognition in Patents (CEMP), (ii) Chemical Passage Detection (CPD) and (iii) Gene and Protein Related Object task (GPRO). Subtask 1, as the main task of this challenge, was a typical NER task. Subtask 2 required participants to identify the sections (the title or abstract) of the patent that contained the chemical. Subtask 3 was to identify mentions of gene and protein related objects. The challenge organizers provided manually annotated abstracts from medicinal chemistry patents (21 000 abstracts in total), of which 7000 abstracts were used as the training set, 7000 abstracts were used as the development set and the remaining 7000 abstracts were used as the test set.

In this article, we describe our systems for the CEMP and CPD tasks. We first used a rule-based module for sentence segmentation and tokenization, and then built machine learning-based NER classifiers using either CRF (31) or structured support vector machines (SSVMs) (32). CRF has been widely used for chemical NER from biomedical literature and patents. SSVM has not been applied to patent text yet, but it has shown the state-of-the-art performance in chemical NER from biomedical literature (14). To evaluate the effectiveness of different types of features, a baseline system was built using a set of common NER features, which have been proved to be effective in different domains (14, 33). Two additional sets of features were then employed to adapt the baseline NER system to patent text: (i) domain knowledge features such as chemical/drug dictionaries, chemical structural patterns and semantic type information present in the context of a candidate chemical and (ii) word representation features generated from large unlabeled corpora by unsupervised learning algorithms including Brown clustering (34) and a novel binarized Word embedding method (35). Such word representation features were assumed to contain latent syntactic/semantic information of a word, thus improving the generalizability of the NER systems. Then we also generated the outputs for the CPD task, by leveraging chemical entities recognized in the patent titles and abstracts by the CEMP task. Our best system achieved the second rank in CEMP with a *F*-measure of 88.94% and the first rank in CPD with a sensitivity of 98.60%, an accuracy of 94.75% and a MCC of 88.24%.

## Materials and Methods

**Figure 1.**
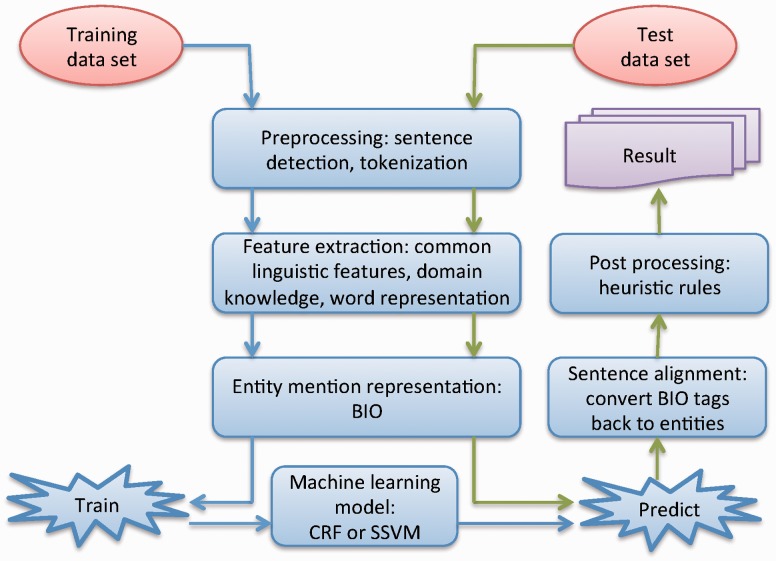
The workflow of our system for chemical named entity recognition from patents.

Figure 1 shows the workflow of our systems for the CEMP subtask, consisting of six components: (i) preprocessing, which breaks a patent document into sentences and tokenizes each sentence using a rule-based approach; (ii) feature extraction, which extracts different types of features from the tokenized sentences; (iii) entity mention representation, which represents entities as a sequence of specific tags; (iv) machine learning model, which uses machine learning algorithms to generate the NER model; (v) sentence alignment, which realigns the predicted tag sequences back to named entities in the original sentence; (vi) post-processing, which uses heuristic rules to reduce errors generated by the machine learning model. The key components of the systems are presented in the following sections in detail.

### Dataset

The organizers collected medicinal chemistry patents from Google patents using International Patent Classification (IPC) code as the selection criteria. A total of 21 000 patent abstracts were manually annotated with seven types of chemical entities based on a pre-defined guideline. The annotated abstracts were divided into three parts: a training set of 7000 abstracts, a development set of 7000 abstracts and a test set of 7000 abstracts. Another 33 000 abstracts formed the test background set that was used to avoid any manual correction of the predictions. We used the training set to build chemical NER systems, which were then validated and tuned using the development set. The optimal systems were finally evaluated on the test set. Table 1 lists the counts of each type of chemical entity in the training and development datasets. The gold-standard annotation of the test dataset has not yet been released by the organizers.

**Table 1.** Statistics of the training and development datasets of the BioCreative V CHEMDNER patents challenge

Types	Training set	Development set
ABBREVIATION	588	454
FAMILY	12 209	11 710
FORMULA	2239	2120
IDENTIFIER	99	125
MULTIPLE	140	141
SYSTEMATIC	9570	9194
TRIVIAL	8698	8298
ALL	32 955	32 042

* CHEMDNER patents: CHEMDNER from patent text.

#### Chemical entity recognition

**Figure 2.**
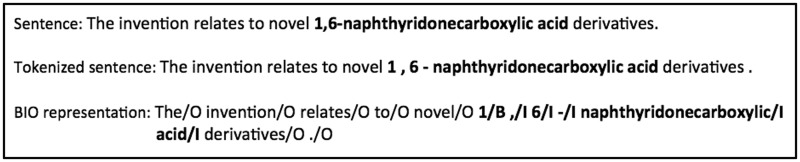
An example of the BIO representation of chemical named entities.

In machine learning-based NER systems, the NER problem is converted into a sequential labeling problem by representing each word using specific labels (36). In our study, we used the BIO labels, a typical representation for named entities, to represent chemical entities, where ‘B’, ‘I’ and ‘O’ denote the beginning, inside and outside of an entity, respectively. Therefore, the chemical entity recognition problem is converted into a sequential labeling problem wherein the task is to assign one of the three labels to each word. Figure 2 shows an example of the BIO representation, where the chemical entity ‘1,6-naphthyridonecarboxylic acid’ is represented as ‘1/B**,/**I 6/I -/I naphthyridonecarboxylic/I acid/I’ after tokenization.

To investigate the effects of features derived from domain knowledge and word representation generated by unsupervised learning in patent text, we first developed a baseline chemical NER system that covers the most common NER features including bag-of-words, orthographic information (word patterns, prefixes and suffixes), syntactic information [POS (part of speech) tags] as well as n-grams of characters, words, POS tags and their combinations (unigrams, bigrams and trigrams) (14). Details of each of the domain knowledge features and unsupervised word representation features used in the systems are presented in the following sections.

#### Features from domain knowledge

Features derived from domain-specific knowledge sources are described below:

Chemical pattern: Features representing characteristics specific to chemicals were adopted from tmChem (37). Furthermore, the annotation guideline for patents also considers general chemical mentions to describe substituents of the general Markush formula that is different from the guideline for scientific literature in BioCreative IV. Hence, the prefix and suffix of chemical functional groups and structural words, such as ‘hydroxyl’, ‘benzyl’ and ‘cyclic’, were also manually collected from the gold book of IUPAC (http://goldbook.iupac.org/) as features of chemical patterns.

The prefixes/suffixes features employed in our systems are defined as the first and last *m* (*m* = 1,2,3) characters of a token; while the n-grams of characters (*n* = 2, 3) are defined as all the n-size contiguous character sequences of a token. Both the features of prefixes/suffixes and n-grams of characters are commonly used in general named entity recognition systems. In contrast, the chemical affix features are used to denote the functional groups or structures of chemicals as domain-specific knowledge. For a more detailed illustration of their differences, Table 2 lists the specific features of each type using the chemical name of ‘benzylamino’ as an example.

**Table 2.** Illustration of features identified as prefixes/suffixes, n-grams of characters and prefixes/suffixes of a chemical named entity

Chemical name	Benzylamino
prefixes/suffixes	b, be, ben, ino, no, o
n-grams of characters	be, ben, en, enz, nz, nzy, zy, zyl, yl, yla, la, lam, am, ami, mi, min, in, ino, no
Prefixes/suffixes of chemicals	benzyl, amino

Gene lexicon: Since many medicinal chemistry patents contain mentions of both chemical compounds and genes/proteins, one type of false positive errors was caused by mistakenly recognizing a gene/protein mention as a chemical compound. Genes and proteins annotated in the training set were used as features to reduce such errors.

**Figure 3.**
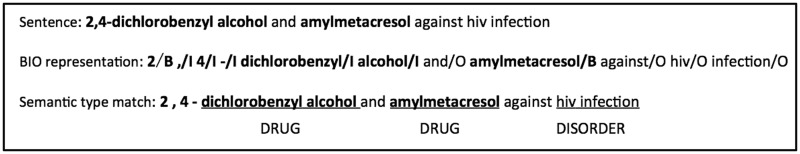
An example of semantic type annotation for context feature extraction.

Semantic type in the Unified Medical Language System (UMLS) (38): Given that NER is a sequential labeling problem, the optimal decision made by the NER system is based on the labels of tokens in the whole sentence. Thus, the global context surrounding a candidate chemical is an important factor to be considered. For this, the semantic type information present in the context of a candidate chemical was generated as a feature by matching the concept terms in UMLS. Figure 3 shows an example of semantic type annotation results.

ChemSpot: The output of the ChemSpot system is used as a feature (11). ChemSpot classifies chemical mentions into different types, similar to the annotation schema of the CHEMDNER patents challenge. The output of ChemSpot was considered as a pre-annotation of chemical entities with BIO tags and chemical types. For example, the feature for ‘hydrocodone*’* was ‘B_ TRIVIAL*’*, instead of a binary feature used in tmChem (37).

#### Unsupervised word representation features

Two types of word representation features were generated from unlabeled patent documents:

Word embedding feature: Word embedding generates a distributional word representation for each word in an unlabeled corpus as a real-valued vector using neural networks (39–41). We used the binarized Word embedding feature proposed in 2014 by Guo et al. (35). The intuition of the binarized embedding feature is to discretize the original real-valued matrix of Word embeddings (41) and omit the insignificant dimensions. Specifically, to convert the real values in the original Word embedding matrix M_V × D_ to discrete symbolic values in [+,−, 0], the positive mean MEAN(j)^+ ^and negative mean MEAN(j)^− ^for the *j*th dimension (column) of M_V × D_ are first calculated as follows (42):

(1) $$\text{MEAN}{\left(j\right)}^{+}=\frac{1}{N_{j}^{+}}\overset{V}{\underset{i=0}{\sum }}M_{i,j},\;M_{i,j}>0$$

(2) $$\text{MEAN}{\left(j\right)}^{-}=\frac{1}{N_{j}^{-}}\overset{V}{\underset{i=0}{\sum }}M_{i,j},\;M_{i,j}<0,\;$$

where *N_j_^+ ^*is the total number of rows with *j*th column *M.j* *>0*, and *N_j_^− ^*is the total number of rows with *j*th column *M.j* *<0.* Then the discrete-valued matrix M*_V × D_ can be derived by the following projection:

(3) $$M_{i,j}^{\text{*}}=\{\begin{array}{c} +,\;if\;M_{i,j}>\text{MEAN}{\left(j\right)}^{+}\\ -,\;if\;M_{i,j}<\text{MEAN}{\left(j\right)}^{-}\\ 0,\;\text{otherwise} \end{array}$$

**Figure 4.**
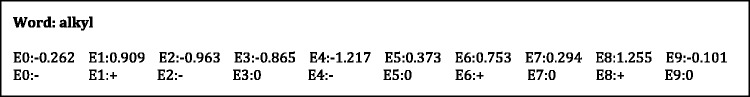
A comparison between real-valued and binarized embedding features.

Values in the $M_{i,j}^{*}$ row of the corresponding word will be used as its Word embedding features. An example of word alkyl in Figure 4 illustrates the difference between the real-valued and binarized features.

#### Brown clustering feature

Brown clustering builds a hierarchical cluster for words in an unlabeled corpus according to the context similarity among those words (34). The hierarchical path of a word in the cluster was used as the word representation feature. We followed the method in (43) to generate Brown clustering features. Specifically, the hierarchical clusters are represented by a binary tree. Words that are semantically/syntactically similar are assumed to be in the same or close clusters and have similar feature representations. For example, both of the words, oxygen and nitrogen are represented as ‘110110110110’ from the hierarchical binary tree generated from 500 Brown clusters.

#### Machine learning algorithms

We investigated two state-of-the-art machine learning algorithms for chemical entity recognition: CRF (31) and SSVM (32).

#### Rule-based post-processing

We also defined some simple rules to fix a number of obvious errors by the machine learning-based classifier. Some examples are listed below:

Conduct a dictionary lookup by exact match in the abstract, using the recognized entities as a lexicon. If there is a string that matches the recognized entity, then label the string as a new entity.

If there is unmatched parenthesis or square bracket in an entity, consider it a false positive and remove it.

If there is a word indicating chemical structure in front of or behind the entity, remove the entity and combine it with the word as a new entity.

### CPD

The CPD task required participants to identify the sections (the title or abstract) of the patent that contained the chemical. We directly leveraged the system output from the CEMP task for CPD. Specifically, the system output for the CPD task was derived based on the patent titles and abstracts with chemicals recognized in the CEMP task.

### Experiments and evaluation

In this study, we started with a baseline system that implemented common features including bag-of-word, orthographic information, morphological information and POS. Then we evaluated the effects of two sets of features: domain knowledge features and unsupervised word representation features, by adding each of them incrementally to the baseline systems. Finally, the post-processing step was added on top of the whole set of features.

The Word embedding features in our study were generated from the deep neural network algorithm, which required a large-scale corpus to tune parameters (39). Considering the challenge time limitation, we used the entire set of abstracts (624, 954) of MedLine with well-formatted text published in 2013 as the corpus to generate Word embedding features. Another participant team of this challenge also used the latest Wikipedia dump as well as the 2013 release of MedLine for Word embedding (44). On the other hand, Brown clustering features were generated from a hierarchical clustering algorithm with relatively less number of parameters (34). Therefore, we directly used the set of abstracts (54 000) provided in the 2015 CHEMDNER patents challenge as the corpus for generating features using Brown clustering. To generate Word embedding features, we implemented the ranking-based deep neural network algorithm according to the paper from Collobert (39) using Java. Parameters suggested in (39) were used to train the neural network with a hidden layer size of 300, a fixed learning rate of 0.01, and an embedding dimension of 50. It took about three weeks to train the Word embedding model. To generate Brown clustering features, we used the implementation from ‘https://github.com/percyliang/Brown-cluster/’ and set the number of clusters to 500. It took about 24 h to generate Brown clusters.

We used CRFsuite (http://www.chokkan.org/software/crfsuite/) and SVMhmm (http://www.cs.cornell.edu/people/tj/svm_light/svm_hmm.html) as implementations of CRF and SSVM, respectively. Their parameters were optimized on the development set while the models were trained on the training set.

Because the gold standard of the test set is not released yet, we report the performance of combining different types of features on the development set. Systems with optimal performance on the development set were submitted to the challenge and officially evaluated by the organizers using the test set. The performance of our systems on the test set is also reported.

The official evaluation portal provided by the CHEMDNER patents organizers were used to calculate the strict micro-averaged precision (*P*), recall (*R*), and *F*-measure for the CEMP task, and sensitivity, specificity, accuracy, Matthew’s correlation coefficient (MCC) and precision at full recall (P_full_R) for the CPD task.

## Results

Table 3 shows the performance of the CRF-based and SSVM-based classifiers on the development set for the CEMP task, with each of the domain knowledge features and unsupervised word representation features added incrementally. The SSVM-based system outperformed the CRF-based system when using the same features. The differences in *F*-measure between them ranged from 0.3% to 0.8%. Each additional feature improved the performance of both the CRF-based and SSVM-based systems. Among the four types of domain knowledge features, the ChemSpot feature contributed maximally to the improvement as compared to the others (CRF: 0.19%, SSVM: 0.20%). Among all the features, the Brown clustering feature contributed the most to the performance improvement (CRF: 0.50%, SSVM: 0.43%). The highest *F*-measures achieved by the CRF-based and SSVM-based systems were 86.96% and 87.74%, respectively. Post**-**processing further enhanced the *F*-measures to 87.22% for the CRF-based system and 87.89% for the SSVM-based system.

**Table 3.** The performance of CRF-based and SSVM-based CEMP systems with different types of features on the development dataset (%)

Method	CRF			SSVM		
	*P*	*R*	*F*-measure	*P*	*R*	*F*-measure
Baseline	85.05	86.18	85.61	85.63	87.78	86.53
+Chemical pattern	85.28	86.16	85.72 (+0.11)	85.82	87.74	86.61 (+0.08)
+Gene lexicon	85.53	86.29	85.91 (+0.19)	85.81	87.92	86.76 (+0.15)
+Semantic type	85.48	86.52	86.00 (+0.09)	85.77	88.27	86.87 (+0.11)
+ChemSpot	82.49	90.24	86.19 (+0.19)	82.86	91.82	87.07 (+0.20)
+Word embedding	82.30	91.06	86.46 (+0.27)	82.73	92.43	87.31 (+0.24)
+Brown clustering	86.34	87.58	86.96 (+0.50)	86.10	89.44	87.74 (+0.43)
+Post**-**processing	86.02	88.45	87.22 (+0.26)	85.88	89.99	87.89 (+0.15)

* CRF: conditional random fields; SSVM: structural support vector machine; CEMP: Chemical Entity Mention Recognition in Patents.

As the systems utilizing all types of features and the post-processing step demonstrated the optimal performance on the development set, we used the same setting for the test set. Performance of the systems for the CEMP task and CPD task on the test set is shown in Tables 4 and 5, respectively. As expected, the SSVM-based system built from the training and development sets achieved the best performance on both tasks. The *F*-measure of 88.94% ranked second among all participating teams for the CEMP task, and the sensitivity of 98.60%, the accuracy of 94.75%, the MCC of 88.24% and the P_full_R of 66.57% ranked first for the CPD task.

**Table 4.** The performance of CRF-based and SSVM-based systems on the test set for the CEMP task (%)

Training dataset	Algorithm	*P*	*R*	*F*-measure
Train + development	CRF	**87.56**	89.64	88.59
Train + development	SSVM	87.18	**90.78**	**88.94**

* CRF: conditional random fields; SSVM: structural support vector machine; CEMP: Chemical Entity Mention Recognition in Patents. Top performance in each column is bolded.

**Table 5.** The performance of CRF-based and SSVM-based systems on the test set for the CPD task (%)

Training dataset	Algorithm	Sensitivity	Specificity	Accuracy	MCC	P_full_R
Train + development	CRF	98.32	**87.27**	94.59	87.85	66.27
Train + development	SSVM	**98.60**	87.21	**94.75**	**88.24**	**66.57**

* CRF: conditional random fields; SSVM: structural support vector machine; CPD: chemical passage detection. Top performance in each column is bolded.

## Discussion

In this study, we conducted a systematic investigation to assess the contribution of different types of features and machine learning algorithms to chemical NER in patents. Experimental results showed that both features generated from domain knowledge and unsupervised learning algorithms made significant improvements to the chemical NER systems for patents. Specifically, the SSVM-based CEMP systems outperformed the CRF-based CEMP systems when using the same features. Our best system based on SSVM achieved the second rank in CEMP with the *F*-measure of 88.94% and the first rank in CPD with the sensitivity of 98.60%, accuracy of 94.75% and MCC of 88.24%, demonstrating the usefulness of the proposed features for chemical NER in patents.

### Comparison between different features

Currently, the ChemSpot system and unsupervised Word embeddings applied in our systems were generated from biomedical literature due to the challenge time limitation. Both of them led to a significant improvement of the recall and a drop in the precision. On the other hand, the Brown clustering features generated from the unlabeled patent corpus created a balance between precision and recall on top of these two features. This indicates that there are similarities between the two genres of literature and patents, as well as fundamental differences. Therefore, features generated from biomedical literature helped to recognize more chemicals, but also increased noise in the system. Overall, unsupervised word representation features contributed a higher performance enhancement than domain knowledge features using the same machine learning algorithms (CRF: 0.58% vs. 0.77%, SSVM: 0.54% vs. 0.67%), demonstrating that unsupervised word representation features have more effective generalization ability over domain knowledge for machine learning-based CEMP systems.

Moreover, additional experiments were conducted after the challenge, to further examine the effect of Word embedding features generated from a large corpus of patents. A new set of Word embedding features were generated using patent abstracts from the United States Patent and Trademark Bulk (USPTO) provided by Google. Patents from the years of 2002 to 2014 were employed, which contained 5 062 891 abstracts in total. Then new experiments were conducted by replacing the original Word embedding features generated from the literature into the new features. By combining domain knowledge features and new Word embedding features, we got an enhanced performance of 86.3% precision, 87.6% recall and 87.0% *F*-measure for the CRF-based system, and 86.2% precision, 89.4% recall and 87.8% *F*-measure for the SSVM-based system. This performance was already comparable to the optimal performance of using all the original features. However, when Brown clustering features were further added, no additional improvement was yielded. This indicated that the contribution from unsupervised word representation features had an upper bound. Adding more features does not necessarily enhance the overall performance continuously.

### Comparison between different machine learning methods

As illustrated in Table 3, SSVM-based system outperformed the CRF-based system in the current NER task, when the same feature sets were used. In our previous studies, we have obtained similar findings on other NER tasks using the same experimental setup for the CRF and SSVM algorithms (14, 33, 45). Moreover, works from other groups also demonstrated the advantage of SSVM over CRF for different sequential labeling tasks, using the same (32, 46) or a different implemental tool for SSVM (47). Specifically, SSVM-based system gained a much higher recall than CRF-based system with a slight sacrifice of the precision, and thus, boosted the overall *F*-measure. The major reason for it was the basic difference between the two algorithms: CRF is a representative sequence labeling algorithm, which is a discriminative undirected probabilistic graphical model (31), whereas SSVM is a large margin-based discriminative algorithm for structural data, such as sequences, bipartite graph and trees (32). By combining the advantages of both CRF and SVM, SSVM demonstrates a stronger generalizability over different NER tasks (14, 33, 45, 47).

### Error analysis

By manually checking the errors generated by our chemical NER system, we found that the major causes of NER errors included (i) mistakenly recognizing gene and protein mentions as chemicals, (ii) breaking long chemicals into multiple chemicals, (iii) recognizing a long chemical partially, (iv) unmatched punctuations of parenthesis and square brackets and (v) missing chemicals from uncommon sentence context. An example for each type of error is listed in Table 6. For errors of type (i), a comprehensive dictionary of genes and proteins could be used to remove such false-positive errors. To correct errors of types (ii)–(v), a partial solution could be the use of post-processing rules as employed in our study. However, more patterns of chemical structures tailored to the patent text should be further explored to improve the performance of our system.

**Table 6.** Examples of chemical named entity recognition errors

Error type	Example
Gene & proteins	The compositions comprise antisense compounds, particularly antisense oligonucleotides, targeted to ***nucleic acids*** encoding C/EBP beta.
Breaking long chemicals	The derivative has a structure expressed by the formula (1), wherein R1 is ***C1 to C11*** chain hydrocarbon
Recognize partially	**Chiral *tricyclic*** compounds with anti-histamine activity
Unmatched punctuations	particularly sphingosine (SPH) and ***sphingosine-1-phosphate (S-1-P)***.
Uncommon context	The invention relates to a steroid derivative which steroidal skeleton is bound at **carbon** atom 17 to a spiromethylene ring of the formula

*The correct chemical mentions are bolded and underlined, while the misrecognized chemicals are bolded and italicized.

For future work, in addition to the error-addressing methods as discussed above, preprocessing tools built specifically for chemical text tokenization need to be investigated, as pointed out in (14). A refined preprocessing module could potentially improve the later stage feature extraction and the final chemical NER performance. Moreover, efforts could be made to further adapt the current features to patent text, such as retraining existing chemical NER systems (e.g., ChemSpot) using the annotated patent corpus.

## Conclusions

In this study, we proposed machine learning-based approaches for the chemical NER task in the CHEMDNER challenge and investigated the contributions of domain knowledge features and unsupervised word representation features. Our systems achieved top-ranked performances in both the CEMP and CPD tasks, demonstrating the effectiveness of the machine learning algorithms (e.g., SSVM) and proposed features in the chemical NER task for patent documents. Currently, an executable package of patent chemical named entity recognition is shared with the community, which could be downloaded from the link: https://sbmi.uth.edu/ccb/resources/patentChemNER.htm.
